# Genotypes in the 17q12‐q21 asthma risk locus and early‐life viral wheezing illnesses

**DOI:** 10.1111/pai.70165

**Published:** 2025-08-04

**Authors:** Nathan Schoettler, Tebeb Gebretsadik, Sweta Singh, Lisa Gress, Eneida A. Mendonça, Brittney M. Snyder, Amy A. Eapen, Petra LeBeau, Ronald Gangnon, Christine M. Seroogy, Leonard B. Bacharier, Robert F. Lemanske, Susan V. Lynch, Diane R. Gold, Rachel L. Miller, Daniel J. Jackson, Gurjit K. Khurana Hershey, Christine C. Johnson, Fernando D. Martinez, Carole Ober, Tina V. Hartert, James E. Gern, the ECHO Children's Respiratory and Environmental Workgroup, Kathrine L. Barnes, Pam Bates, Jessica Baucom, Patrice Becker, Casper G. Bendixsen, Alex Binder, Nikki Bires, Rashida Blackwood, Yury A. Bochkov, Steven M. Brunwasser, Richard Budrevich, Emily Bull, Thomas Callaci, Kirsten Carlson‐Dakes, Tara F. Carr, Jacqueline Cephus, James D. Chappell, Jack Charles, Deborah Chasman, Teresa M. Chipps, Tatiana Chirkova, Kamry Coffey, Gina Crisafi, Suman R. Das, Douglas DaSilva, Lydia De La Ossa, Stephanie Doss, Amy Dresen, William D. Dupont, Abby Engelhart, Heather Floerke, Wais Folad, Terry Foss, Angela Freie, Wayne Frome, Samantha Fye, Lisa Gagalis, Peter Gergen, Nicole Gonzalez, Kayla Goodman, Kristine Grindle, Brian Hallmark, Marilyn Halonen, Erin Higdon, Samadhan J. Jadhao, Molly Johnson, Tara Johnson, Meyer Kattan, Rick Kelley, Tammy Koepel, Cole Lacey, Laura Ladick, Carin Lamm, Kristine Lee, Stephanie Leimenstoll, Zhouwen Liu, Silvia Lopez, Stephanie Lovinsky‐Desir, Ana Manuelian, Jennifer Markevich, Lisa Martin, Jomol Matthew, Christopher G. McKennan, Jennifer Meece, Lance Mikus, Vicki Moon, Wayne J. Morgan, George T. O’Connor, Brent F. Olson, Irene Ong, Tressa Pappas, Barron Patterson, Brenda Patterson, R. Stokes Peebles, Marcela Pierce, DeeAnn Polacek, Kadijah Poleon, Victoria Rajamanickam, Kimberly Ray, Sara Reiss, Chris M Reyes, Kathleen Roberg, Christian Rosas‐Salazar, Pat Russell, Lisa Salazar, Hugh Sampson, Megan T. Sandel, Ruchika Sangani, Dena Scott, Meghan H. Shilts, Akihiro Shiroshita, Gina Simpson, Zhengzheng Tang, Christopher Tisler, Alkis Togias, Jeffrey J. VanWormer, Renee Welch, Robert A. Wood, Anne L. Wright, Rosalind J. Wright, Melissa Yaeger, Perri Yaniv

**Affiliations:** ^1^ Department of Medicine University of Chicago Chicago Illinois USA; ^2^ Department of Biostatistics Vanderbilt University Medical Center Nashville Tennessee USA; ^3^ Clinical and Health Informatics Institute (CHI2), School of Medicine and Public Health, University of Wisconsin Madison Wisconsin USA; ^4^ Department of Pediatrics University of Wisconsin School of Medicine and Public Health Madison Wisconsin USA; ^5^ Department of Pediatrics University of Cincinnati College of Medicine and Cincinnati Children's Hospital Medical Center Cincinnati Ohio USA; ^6^ Department of Medicine and Pediatrics Vanderbilt University Medical Center Nashville Tennessee USA; ^7^ Division of Allergy and Clinical Immunology, Department of Internal Medicine Henry Ford Health Detroit Michigan USA; ^8^ Rho, Inc. Federal Research Operations Durham North Carolina USA; ^9^ Department of Biostatistics and Medical Informatics and Department of Population Health Sciences University of Wisconsin School of Medicine and Public Health Madison Wisconsin USA; ^10^ Division of Pediatric Allergy, Immunology and Pulmonary Medicine Monroe Carell Jr Children's Hospital at Vanderbilt Nashville Tennessee USA; ^11^ Benioff Center for Microbiome Medicine, Department of Medicine University of California San Francisco California USA; ^12^ Channing Division of Network Medicine, Department of Medicine Brigham and Women's Hospital Boston Massachusetts USA; ^13^ Department of Environmental Health, Harvard T.H. Chan School of Public Health Harvard University Boston Massachusetts USA; ^14^ Division of Clinical Immunology, Department of Medicine Icahn School of Medicine at Mount Sinai New York New York USA; ^15^ Cincinnati Children's Hospital, Division of Asthma Research Cincinnati Ohio USA; ^16^ Department of Public Health Sciences Henry Ford Health Detroit Michigan USA; ^17^ Asthma and Airway Disease Research Center and Division of Pulmonary and Sleep Medicine, Department of Pediatrics College of Medicine, University of Arizona Tucson Arizona USA; ^18^ Department of Human Genetics University of Chicago Chicago Illinois USA

**Keywords:** 17q12‐q21, asthma, childhood, respiratory syncytial virus, rhinovirus, viral wheezing

To the Editor,

Infections with rhinovirus (RV) or respiratory syncytial virus (RSV) are the two most common triggers of wheezing illnesses in preschoolers. These illnesses are leading causes of hospitalization in the early years and also represent a significant risk factor for developing childhood asthma.^1^, ^2^ While lower respiratory illnesses caused by RSV are preventable, there are no vaccines or antivirals available for RVs. This therapeutic gap highlights the critical need to identify the pathogenic mechanisms behind virus‐induced wheezing illnesses and to develop new strategies for treatment or prevention.

Genetic, environmental, and personal factors contribute to the risk of preschool wheezing illnesses. These include genes that regulate immune responses and cell‐surface receptors utilized by viruses. A genomic region on chromosome 17q12‐q21, which encodes the genes *ORMDL3* and *GSDMB*, significantly increases the risk for viral wheeze.^3^ Interestingly, this region is also the most significant and replicated locus for childhood‐onset asthma,^4^, ^5^ especially in children with a history of wheeze^6^ and/or RV wheeze.^3^ In a combined analysis of two birth cohorts, it was unable to resolve whether relationships between 17q12‐q21 genotype, viral wheezing illnesses, and childhood asthma depended on the virus causing the initial wheezing episodes.^3^ Another limitation of previous studies is that they were conducted in children with genetic ancestry most similar to European populations.

We sought to address these limitations by investigating the relationships between genetic variation at the 17q12‐q21 locus and early‐life viral wheezing illness in children from four birth cohorts participating in the Children's Respiratory and Environment Workgroup (CREW),^7^ a consortium funded by the NIH's Environmental Influences on Child Health Outcomes (ECHO) program.^8^ These children are diverse with respect to ancestry, geography, and socio‐demographic factors associated with asthma. We tested for associations between single nucleotide polymorphisms (SNPs) across the extended 17q12‐q21 region and time to RV‐ and RSV‐specific wheezing illness and analyzed the role of parent‐reported race.

The study population consisted of 1475 children enrolled in four birth cohorts: the Tucson Children's Respiratory Study (TCRS), the Childhood Origins of Asthma study (COAST), the Urban Environment and Childhood Asthma (URECA) study, and the Infant Susceptibility to Pulmonary Infections and Asthma Following RSV Exposure (INSPIRE) study (Table 1). This work was approved by the institutional review boards at the participating institutions. SNPs were genotyped using a TaqMan assay as previously reported.^9^ Because of the strong LD among SNPs within each of the three regions,^9^ we selected one SNP from each region as a surrogate for other variants in those regions. To this end, we selected rs2941504 in the proximal region because it showed the least LD with the core region SNPs in CREW children who identified as White or Black^9^ and was an eQTL for *PGAP3*.^10^ We selected rs7216386 in the core region because it was previously associated with RV wheezing illness and is an eQTL for *GSDMB* and *ORMDL3*.^3^ In the distal region, we selected rs3859192 because it had the least LD with the core region SNPs^9^, ^11^ and was an eQTL for *GSDMA*.^12^ A parent or guardian provided written informed consent for their child. Parent‐identified Black children comprised 32.1% of the subjects. RV wheezing illnesses occurred in 19.2% of children identified as White and 42.8% identified as Black. RSV wheezing illnesses occurred in 21.4% and 16.7% of children identified as White and Black, respectively.

**TABLE 1:** Participating CREW Cohorts*.

Cohort	Children parent‐identified as White				Children parent‐identified as Black			Overall
	COAST	INSPIRE	TCRS	Total (%)	INSPIRE	URECA	Total (%)	
Subjects included, *N* ^a^	215	394	393	1002 (67.9%)	98	375	473 (32.1%)	1475
Sex (% Boys)	57.7	52.8	50.9	53.1	54.1	51.5	52.0	52.8
RV wheezing events	62 (28.8%)	55 (14.0%)	NA	117 (19.2%)	14 (14.3%)	186 (49.6%)	200 (42.8%)	317 (29.3%)
RSV wheezing events	65 (30.2%)	88 (22.3%)	61 (15.5%)	214 (21.4%)	21 (21.4%)	58 (15.5%)	79 (16.7%)	293 (19.9%)

Abbreviations: COAST, Childhood Origins of Asthma Study; INSPIRE, Infant Susceptibility to Pulmonary Infections and Asthma Following RSV Exposure study; NA, not applicable; RSV, respiratory syncytial virus; RV, rhinovirus; TCRS, Tucson Children's Respiratory Study; URECA, Urban Environment and Childhood Asthma study; WISC, Wisconsin Infant Study Cohort.

^a^ Subjects included in the analysis had 17q12‐q21 genotyped, the ascertained status of RV or RSV wheezing with age up to 3 years, parent‐identified race as White or Black.

To investigate genotype effects on time to first viral wheezing illness in early life, we performed time‐to‐event analyses separately for RV and RSV wheezing illnesses during the first 3 years of life. We stratified this analysis by race due to differences in genetic architecture in this region. European ancestry is associated with extensive linkage disequilibrium, while African ancestry is not and is associated with unique haplotypes. In the 17q12‐q21 core region, the rs7216389 TT genotype has been associated with increased risk of childhood asthma.^5^ In White children, rs7216389‐T was associated with more children having RV wheezing events (*p*‐value = .006; Figure 1A). For every increase in the number of T alleles (compared with the CC genotype), RV wheezing illness risk increased 46% (Cox model hazard ratio [HR] 1.46; 95% confidence interval [CI] = 1.16–1.85, Table 2). In the Black children, time to RV wheezing events was not significantly associated with rs7216389 genotype (rs7216389: HR 1.08, 95% CI = 0.83–1.40; Figure 1B). No associations were observed with rs2941504 or rs7216386.

**FIGURE 1:**
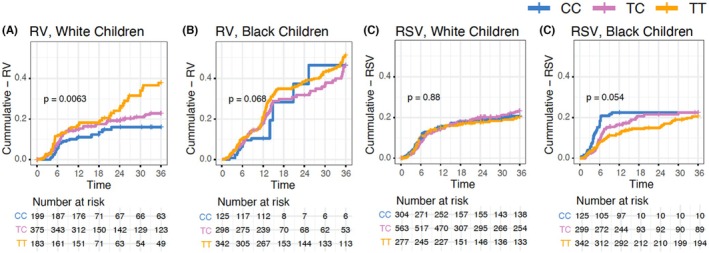
Time‐to‐viral wheezing illness and 17q12‐q21 genotypes. Kaplan–Meier curves for time to wheezing illness for the core‐rs7216389 SNP. Separate plots are shown for results stratified by virus and parent‐reported race. *p* values are from the log‐rank test. The numbers of children in each group at each timepoint are shown at the bottom of each panel.

**TABLE 2:** Cox model hazard risk^a^ for time from birth to RV or RSV wheezing illness by rs7216389 T genotype.

Analysis	Hazard ratio	95% confidence interval	*p*‐Value
RV – White	1.464	1.157–1.851	<.001
RV – Black	1.080	0.831–1.404	.572
RSV – White	0.984	0.816–1.185	.868
RSV – Black	1.250	0.804–1.944	.302

^a^ Cox proportional hazard model included child sex, birth month, and cohort.

For RSV wheezing illnesses, there was no association with rs7216389 genotype in White children (Figure 1C), while in Black children there was a nonsignificant trend for rs7216389‐TT asthma‐risk genotype having a longer time‐to‐RSV wheezing illness (*p*‐value .054, Figure 1D). Neither the proximal‐rs2941504 nor the distal‐rs3859192 region SNP was associated with RV or RSV wheezing illnesses in either White children or Black children (data not shown).

Previous studies have shown that variants at the 17q12‐q21 locus are strongly associated with preschool wheezing illnesses, wheezing phenotypes, early‐onset asthma, and asthma exacerbations and hospitalizations before the age of 5 years (reviewed in^13^). Furthermore, genotypes at the 17q12‐q21 locus can modify the effects of environmental exposures, including adverse effects of environmental tobacco smoke and beneficial effects of older siblings,^6^ farm animals, and pets.^14^

Our findings add to this growing literature by evaluating virus species and self‐reported race as modifying factors across multiple birth cohorts. We showed that the rs7216389 asthma‐risk allele of variation in the 17q12‐q21 core region, but not in the proximal or distal regions, was associated with the time to first RV wheezing illness in preschoolers. These associations were significant for children identified as White for RV, but not significant for children identified as Black.

In contrast, the rs7216389 asthma‐risk allele tended to be associated with a shorter time to first RSV wheeze in Black‐ and White‐identified children. These effects may relate to differences in the pathogenesis of wheezing episodes caused by RV compared to RSV. For example, a recent study demonstrated that the rs7216389 asthma‐risk genotype is associated with lower expression of interferon‐inducible antiviral cytokines.^15^ Because the RSV NS1 and NS2 proteins markedly inhibit interferon responses,^16^ reduced interferon responses due to genetics could be a more substantial risk factor promoting severe RV illnesses.

The strengths of our study include using information from four different cohorts that include a broad demographic, availability of viral diagnostics, and prospective study designs that enabled consideration of multiple predictors and covariates. There are also limitations to consider. Using the parent‐identified racial categories does not accurately capture ancestry effects but is likely to reflect exposures that correlate with identified race, including both physical and sociocultural environments. One potential explanation for the difference between these groups of children is that the precision of rs7216389 tagging the true causal variant at this locus may differ due to the different LD patterns between individuals of European and African ancestries.^5^

In conclusion, we demonstrated that the asthma risk allele at the 17q12‐q21 core region is related to RV wheezing illnesses. The association with RSV, while of borderline significance, was in the other direction. These findings suggest that the pathogenic mechanisms of RV and RSV wheezing illnesses have distinct features. Given the lack of preventive treatments for RV, identifying mechanisms for RV wheezing illnesses could be an important step toward novel therapeutics.

## AUTHOR CONTRIBUTIONS

**Nathan Schoettler:** Conceptualization; formal analysis; writing – original draft; visualization; writing – review and editing; investigation. **Tebeb Gebretsadik:** Formal analysis; visualization; writing – review and editing; data curation. **Sweta Singh:** Writing – review and editing; data curation. **Lisa Gress:** Writing – review and editing; data curation. **Eneida A. Mendonça:** Writing – review and editing. **Brittney M. Snyder:** Writing – review and editing. **Amy A. Eapen:** Data curation; writing – review and editing. **Petra LeBeau:** Data curation; writing – review and editing. **Ronald Gangnon:** Writing – review and editing. **Christine M. Seroogy:** Data curation; writing – review and editing. **Leonard B. Bacharier:** Writing – review and editing. **Robert F. Lemanske Jr:** Conceptualization; writing – review and editing. **Susan V. Lynch:** Writing – review and editing. **Diane R. Gold:** Data curation; writing – review and editing. **Rachel L. Miller:** Writing – review and editing; data curation. **Daniel J. Jackson:** Data curation; writing – review and editing. **Gurjit K. Khurana Hershey:** Writing – review and editing. **Christine C. Johnson:** Writing – review and editing. **Fernando D. Martinez:** Data curation; writing – review and editing. **Carole Ober:** Conceptualization; writing – review and editing. **Tina V. Hartert:** Data curation; writing – review and editing. **James E. Gern:** Conceptualization; data curation; writing – review and editing.

## FUNDING INFORMATION

N.S.: NIH K08 HL153955, U19 AI62310; C.M.S.: U19AI104317; D.R.G.: P30‐ES000002; R.L.M.: UG3/UH3OD023290; D.J.J.: UM1 AI114271, UM1 AI160040; C.O.: U19 AI62310, UM1 AI160040; T.H.: U19 AI 095227, UL1 TR002243. Research reported in this publication was supported by the CREW consortium, Office of the Director, National Institutes of Health under Award Number UH3 OD023282, and CADRE under Award Number U24AI179612.

## CONFLICT OF INTEREST STATEMENT

L.B.B. is a member of the GINA Science Committee; reports grants from NIH/NIAID/NHLBI, personal fees from GlaxoSmithKline, Genentech/Novartis, Merck, Teva, Boehringer Ingelheim, AstraZeneca, Avillion, WebMD/Medscape, Sanofi/Regeneron, Vectura, Circassia, OM Pharma, Recludix, and Kinaset; for DSMB from AstraZeneca, DBV Technologies, Aravax, and Vertex; and royalties from Elsevier outside the submitted work. S.V.L. serves on the board of directors for Siolta Therapeutics Inc. and holds stock options for this company. D.J.J. reports grants from NIAID/NHLBI, GSK, and Regeneron. Personal fees for consulting from AstraZeneca, Avillion, Genentech, GSK, Regeneron, Sanofi, and DSMB for Pfizer and AstraZeneca. T.V.H. serves as the Chair of the vaccine and immunization initiative for the American Thoracic Society and on a Data Safety Monitoring Board for respiratory syncytial virus vaccines for Pfizer and reported grants from NIH and the World Health Organization and Bill and Melinda Gates Foundation during the conduct of the study and personal fees from the NIH, Parker B. Francis Foundation Council of Scientific Advisors, American Thoracic Society, Pfizer, and Sanofi outside the submitted work. J.E.G. reports grants from NIH during the conduct of the study, personal fees from Arrowhead Pharmaceuticals, AstraZeneca, and Meissa Vaccines Inc., and stock options for Meissa Vaccines Inc. outside the submitted work. No other conflicts reported by other authors.

## PEER REVIEW

The peer review history for this article is available at https://www.webofscience.com/api/gateway/wos/peer‐review/10.1111/pai.70165.
